# Arthropod-Borne Pathogens in Wild Canids

**DOI:** 10.3390/vetsci10020165

**Published:** 2023-02-19

**Authors:** Valentina Virginia Ebani, Simona Nardoni, Francesca Mancianti

**Affiliations:** 1 Department of Veterinary Sciences, University of Pisa, Viale delle Piagge 2, 56124 Pisa, Italy; 2 Centre for Climate Change Impact, University of Pisa, Via del Borghetto 80, 56124 Pisa, Italy; 3 Interdepartmental Research Center “Nutraceuticals and Food for Health”, University of Pisa, Via del Borghetto 80, 56124 Pisa, Italy

**Keywords:** wild canids, Anaplasmataceae, *Borrelia* spp., *Bartonella* spp., *Rickettsia* spp., *Leishmania* spp., *Hepatozoon* spp., piroplasms

## Abstract

**Simple Summary:**

Wild canids are largely exposed to ticks and other hematophagous vectors that can transmit numerous bacterial and protozoal pathogens. In the last years, also because of climatic changes, the incidence of arthropod-borne diseases has notably increased becoming a serious threat for humans and animals. Main information about these infections concerns domestic and companion animals, whereas data about their spread among wild canids and their impact on health status of these animals are not exhaustive. Otherwise, studies about bacterial and protozoal arthropod-borne infections in wild canids are important to better understand the risk of infections for dogs and humans.

**Abstract:**

Wild canids, as well as other wild animal species, are largely exposed to bites by ticks and other hematophagous vectors where the features favoring their presence and spread are found in wooded and semi-wooded areas. Much of the information about arthropod-borne infections concerns domestic and companion animals, whereas data about these infections in wild canids are not exhaustive. The present study is a narrative review of the literature concerning vector-borne infections in wild canids, highlighting their role in the epidemiology of arthropod-borne bacteria and protozoa.

## 1. Introduction

In the last years, we have assisted in the increasing incidence of arthropod-borne diseases in humans and animals, in Europe [1] and worldwide [2,3,4,5]. Climate changes, the spreading of wild animals and the animal trade are the main causes of the spread of arthropod vectors across the world, from endemic areas to previously not colonized environments. Wild and domestic carnivores are considered the primary sources of arthropod-borne zoonotic agents to humans. Wild canids, as well as other wild animal species, are largely exposed to bites by ticks and other hematophagous vectors (fleas, lice, mosquitos) where the features favoring their presence and spread are found in wooded and semi-wooded areas. Consequently, arthropod-borne pathogens are largely circulating among wildlife which can reach domestic animals and humans.

Much of the information about arthropod-borne infections concerns domestic and companion animals [6]. In fact, probably because of the difficulty to obtain biological samples, few studies have been performed to investigate the epidemiological and pathogenic aspects of these pathogens in wild canids.

The present study is a narrative review of the literature concerning vector-borne infections in wild canids, highlighting their role in the epidemiology of arthropod-borne bacteria and protozoa.

## 2. *Ehrlichia canis*

*Ehrlichia canis* is a Gram-negative, obligate intracellular bacterium targeting monocytes and belonging to Anaplasmataceae family, order Rickettsiales [7]. It is mainly transmitted by the brown dog tick *Rhipicephalus sanguineus* and it is known as the primary etiologic agent of the canine monocytic ehrlichiosis worldwide. The prevalence and severity of this infectious disease is related to the strain, concurrent infections, host immune reaction, and clinical phase of the disease. Dogs develop acute or chronic disease characterized by fever, depression, lethargy, anorexia, lymphadenomegaly, splenomegaly and hemorrhagic tendencies (usually exhibited by dermal petechiae and ecchymoses, and epistaxis). Ophthalmological lesions such as anterior uveitis, chorioretinitis, papilledema, retinal hemorrhage, presence of retinal perivascular infiltrates and bullous retinal detachment are often present [8]. *Ehrlichia canis* has been suggested as a potential zoonotic agent because some cases of human monocytic ehrlichiosis caused by this pathogen have been reported in Venezuela [9], Costa Rica [10] and Panama [11].

Although the majority of studies about *E. canis* infection have been carried out in dogs because of its important clinical impact on these animals, some researchers investigated the susceptibility of wild canids to this bacterial agent.

In 1938, Neitz and Thomas suggested that the wild dog (*Lycaon pictus)* may act as a reservoir for *E. canis* in the Kruger National Park in Africa, and reported that jackals can be infected [12]. Successively, in 1964, Ewing and collaborators showed, through an experimental infection, the susceptibility of coyotes (*Canis latrans*) to *E. canis* [13]. In 1973, Amyx and Huxoll experimentally infected red (*Vulpes fulva*) and gray (*Urocyon cinereoargeneus*) foxes with *E. canis*; foxes developed mild anemia, thrombocytopenia, and leukopenia in the acute stages of the disease, along with a rise in erythrocyte sedimentation; typical morulae of *E. canis* were demonstrated in Giemsa-stained venous blood smears from the second week through the fifth week post inoculation [14].

At the end of the 1970s, the susceptibility of wolves to *E. canis* was also demonstrated. In fact, Harvey and collaborators [15] diagnosed *E. canis* infection in wolves, dogs and wolf–dog crosses at a small zoo in Northcentral Florida (USA). They observed that *E. canis* was able to cause disease in all of them; in fact, five of nine adult canids and all pups confined to a common kennel died because of the infection. Hematologic and pathologic findings in an adult wolf–dog cross that died were characteristic of canine ehrlichiosis. Moreover, that animal and four other canids had antibodies to *E. canis.* The epizootic was associated with a massive *R. sanguineus* infestation [15].

More recently, wolves have been found susceptible to *E. canis* infection in Europe as well. *Ehrlichia canis* was detected in three out of six gray wolves (*Canis lupus*) analyzed in Southern Italy, other than in 55 out of 105 (52%) red foxes (*Vulpes vulpes*) [16]. Furthermore, a 11.43% prevalence for Anaplasmataceae was found testing spleens from 33 wolves (*C. lupus*) in Northwest Italy, but none of these positive samples were sequenced due to the poor quality of the amplicons, so it was not possible to determine if *Ehrlichia* sp. or *Anaplasma* sp. was responsible for the infection. During the same survey, a 10.97% prevalence was detected in red foxes for Anaplasmataceae microorganisms [17].

Recently, maned wolves (*Chrysocyon brachyurus*), highly infested by *Amblyomma tigrinum*, were tested in Brazil; all the 37 blood samples submitted to molecular analyses resulted in being PCR negative for *E. canis*, whereas 36% (16/45) of the serum samples analyzed by the immunofluorescent test had antibodies against the pathogen [18].

The circulation of *E. canis* among wild canids in Brazil had been previously demonstrated. In particular, in Pantanal, a 17.9% (14/78) seroprevalence to *E. canis* antigen was found among *Cerdocyon thous* foxes and *Ehrlichia* spp. DNA was detected in two *C. thous* [19]. Furthermore, previous studies had already detected *Ehrlichia* spp. DNA, genetically related to *Ehrlichia chaffeensis* and *E. canis*, among wild canids (*C. thous*, bush dog—Speothos venaticus and *C. lupus*) maintained in captivity in Brazilian zoos [20] and among road-killed *C. thous* in the state of Espírito Santo, Southeast Brazil [21]. These findings suggested that crab-eating foxes could be exposed to *E. canis* as well as other ehrlichial species. However, it is worth noticing that while *Ehrlichia* spp. DNA has been detected among Brazilian canids, no clinical signs suggestive of ehrlichiosis have been reported until now in this group of wild mammals.

Jackals have been proven to be susceptible to *E. canis* as well. Price and collaborators [22] isolated *E. canis* in eight out of sixteen free-living jackals (*Canis mesomelas*) in Kenya and experimentally reproduced the infection in two cross-bred puppies, which developed mild disease, through the inoculation of blood from infected jackals.

Moreover, high seroprevalence rates, 35.8% (19/53) and 54.3% (25/46), for *E. canis* were detected in free-ranging Israeli golden jackals (*Canis aureus*) in two surveys, respectively, carried out about 10 years apart, suggesting the increasing spread of the pathogen among jackals in Israel [23,24].

Foxes are the wild canids most largely present in several geographical areas and most studies about infections by canid arthropod-borne bacteria have been carried out in these animals.

Serological evidence of exposure to *E. canis* in red foxes (*V. vulpes*) was found in Israel, where 36% (30/84) of sera examined samples were reactive to *E. canis* antigen [25]. In Switzerland, none of the 1550 red fox sera samples tested for *E. canis* were positive [26], whereas 6.7% (4/60) of a red fox population in the Netherlands had detectable antibodies against monocytic ehrlichia (*E. chaffeensiis*) [27]. Similarly, molecular surveys carried out in different European areas found *E. canis* positivity rates very variable in relation to the investigated area and the years of sampling: 2.9% (2/69) in Portugal [28], 16.6% (2/12) in Spain [29], 52% (55/105) in 2012–2015 [16] and 2.9% (7/244) in 2017–2019 [30] in Southern Italy, 0% in 2007–2008 (0/150) and 2016–2021 (0/22) [31,32] and 44.4% (68/153) in 2014–2016 in Central Italy [33].

A new member of the family Anaplasmataceae was first described in red foxes from Austria and identified as Candidatus Neoehrlichia sp. [34]. This new member has been also detected in a fox from the Czech Republic [35] and in a European badger from Hungary [36]. The circulation of this new agent has been recently reported also by Sgroi et al. [30] in Southern Italy and Lesiczka et al. [37] in the Czech Republic which found 1.2% (3/244) and 3.42% (4/117) red foxes PCR positive for Candidatus Neoehrlichia sp., respectively. Vectors and pathogenic properties of this new strain have not been verified. In addition, it is interesting the detection in a red fox of DNA of an unidentified Ehrlichia sp. strain with unclear phylogenetic position [37].

## 3. *Anaplasma phagocytophilum*

*Anaplasma phagocytophilum* (family Anaplasmataceae, order Rickettsiales) is a tick-borne obligate intracellular bacterium that infects vertebrate host granulocytes, mainly neutrophils, causing human, canine and equine granulocytic anaplasmosis and tick-borne fever of ruminants [7]. It is mainly transmitted by *Ixodes* ticks of which the major vector species are *I. scapularis* in the USA and *I. ricinus* in Europe [38]. *Anaplasma phagocytophilum* has been largely investigated in canine populations worldwide, whereas reports about the exposure of wild canids to this pathogen are less numerous. This bacterial species is largely widespread among wildlife and wild mammals such as squirrels, voles, white-footed mouse, wood rats, white-tailed deer, red deer, and roe deer, have been identified as reservoirs [38].

The first case of granulocytic anaplasmosis in a wolf was reported by Leschnik et al. in 2012 who suggested that *A. phagocytophilum* might cause clinical disease in this animal species [39]. In particular, it was described a clinical case of a 2-year-old male captive timber wolf (*Canis lupus occidentalis*), kept in an outdoor enclosure in Austria, with anorexia, depression and fever. Tick infestation was reported despite monthly acaricidal treatment. The microscopic examination of a blood smear revealed elementary bodies and morulae suspicious for *A. phagocytophilum*. PCR analysis confirmed the infection by the G-variant of the pathogen, also found in horses and humans but not in wild ruminants. The wolf had specific antibodies and it recovered after therapy with doxycycline for 10 days. The clinical status, including the temporary changes in blood parameters (thrombocytopenia, lymphopenia, mild anemia) showed high similarity to granulocytic anaplasmosis usually observed in dogs [39]. 

Successively, the first case of anaplasmosis in wolves in Europe has been reported as well. Two free-ranging gray wolves (*C. lupus*) living in Germany were found PCR positive for *A. phagocytophilum*, even though clinical parameters were not observed [40].

Coyotes (*C. latrans*) have been investigated in California, USA: all tested animals were seronegative for *E. canis* whereas a 46% (68/149) seroprevalence for *A. phagocytophilum* was detected; one seropositive coyote also resulted PCR positive to the agent and sequencing analysis revealed full homology to *A. phagocytophilum* strains circulating in California [41].

Regarding jackals, a study described the detection of a relatively high prevalence (14/53; 26.4%) of antibodies against A. phagocytophilum in C. aureus syriacus in Israel [23], whereas a molecular investigation detected the pathogen in 0.9% (2/216) of the analyzed spleens from jackals in Serbia [42]. In addition, *Anaplasma* sp. closely related to *A. phagocytophilum* has been detected in black-backed jackals (*C. mesomelas*) in South Africa (82/142; 57.7%) [43].

Red foxes (*V. vulpes)* have been investigated in different European areas, where they are largely present. Pusterla et al. [26] found 2.8% (44/1550) seroprevalence for *A. phagocytophilum* among red foxes in Switzerland. Moreover, different molecular prevalence rates have been detected: from 0.6% (1/153) to 16.6% (25/150) in Italy [31,32,33], 0.6% (3/506) in Austria [44], 2.4% (4/162) in Switzerland [45], 2.55% (9/353) in Romania [46], 2.56% (3/117) [37] and 4% (1/25) [47] in the Czech Republic, 2.7% (3/111) in Poland [48], 8.2% (10/122) in Germany [49], 9.9% (8/81) in the Netherlands [50] and 12.5% (52/415) in Hungary [51].

*A. phagocytophilum* has also been detected in gray foxes (*U. cinereoargenteus*); a 51% (36/70) seroprevalence was found in gray foxes in Northern California, USA, whereas only 9% (6/70) of the same animals resulted PCR positive [52]. *Ixodes pacificus, Ixodes texanus, Dermacentor variabilis* and *Dermacentor occidentalis* were identified on the sampled foxes, but no molecular analyses were executed so it was not possible to verify if all these tick species are involved in the transmission of *A. phagocytophilum* [52].

## 4. *Borrelia burgdorferi* sensu lato

*Borrelia burgdorferi* sensu lato (s.l.) is the etiologic agent of Lyme disease, a well-known disease clinically described for the first time in 1977 in the USA and affecting mainly humans which determines severe clinical signs characterized by cutaneous rash (erythema chronicum migrans), fever, headaches, myalgia, stiff neck, arthralgia, and lymphadenopathy; hearth and nervous system involvement can occur in untreated patients [53].

It belongs to Spirochaetales order, Borreliaceae family and includes *B. burgdorferi* sensu stricto (s.s.), *B. garinii* and *B. afzelii* [54]. Lyme disease in the USA is caused by the single species *B. burgdorferi* s.s., whereas in Europe and Asia all the three species are responsible for most human cases of disease [55,56]. The main vectors are ticks of *Ixodes* genus: *I. ricinus* (European sheep tick) in Europe, *I. persulcatus* (taiga tick) in Asia, *I. scapularis* (black-legged tick) in the Northeastern and Midwestern United States, *I. pacificus* (Western black-legged tick) in Western USA [56].

*B. burgdorferi* s.l. is currently widespread in America and Europe. In view of the relevant zoonotic impact, many surveys were carried out to verify prevalence and infected hosts and high circulation of this pathogen in wildlife was observed. For this reason, attention was turned also to wild canids. Kazmierczak et al. [57] carried out an experimental infection in a gray wolf (*C. lupus*) through intravenous injection of a *B. burgdorferi* strain previously isolated from the blood of a *Peromyscus leucopus* trapped at Ft. McCoy, Wisconsin (USA). The wolf developed generalized lymphadenopathy and an antibody titer of 1:512 until 75 p.i. day when it was euthanized; spirochetes were visualized in liver sections by direct immunofluorescent staining. Another wolf, subcutaneously inoculated, showed a low and transient antibody response which peaked at 1:64, and manifested no clinical or postmortem abnormalities.

The same authors detected antibodies against *B. burgdorferi* in 3% (2/78) of gray wolves captured in Minnesota and Winsconsin from 1977 and 1984, showing natural exposure of wolves to this microorganism [57]. Similarly, a 2.5% (15/589) seroprevalence was found by Thieking et al. [58] testing gray wolves from the same areas from 1972 to 1989.

A most recent survey found a seroprevalence of 65.6% (244/372) for *B. burgdorferi* in a wolf population in Wisconsin between 1985 and 2011 [59]. Strong evidence of a positive association of gray wolf coexposure to *B. burgdorferi* and *A. phagocytophilum* was observed. This association is not surprising, because these pathogens share vectors and reservoir hosts (rodents). No data about clinical signs due to *B. burgdorferi* in naturally infected wolves are available and the potential impact of coinfection/coexposure has not been studied in these animals. However, it has been supposed that, as it occurs in other animal species, the intensity and duration of *B. burgdorferi* infection can increase because of the immunosuppressive action of *A. phagocytophilum* [59].

Several red wolves (*C. rufus*) housed at the Great Smoky Mountains National Park, USA, were serologically positive for B. burgdorferi; one positive wolf also exhibited clinical signs, including decreased appetite, weight loss and carpal lesions [60].

Other wild canids may be infected by *B. burgdorferi*. Between 1990 and 2006, serum samples were obtained from 34 wolves and 84 red foxes (*V. vulpes*) throughout mainland Spain. Twelve sera tested positive for the presence of antibodies to *B. burgdorferi*, seven (8.3%) of them from red foxes and five (14.7%) from wolves. Seropositive animals were recorded in the northern areas of Spain where *I. ricinus* was largely present and *B. burgdorferi* was considered endemic [61].

*B. burgdorferi* s.l. has been proven to infect foxes in other geographic areas. In Norway only one red fox, among six tested, was found PCR positive for *B. bugdorferi* s.l. [62], whereas in a study carried out in Germany 24% of skin samples from red foxes had *B. garinii* DNA [63]. Dumitrache et al. [46] found *B. afzelii* and *B. burgdorferi* s.s. in 1.4% (5/353) of analyzed fox hearts in Romania. Moreover, recently, 23.5% (57/243) of red foxes from west-central Poland resulted in being positive for *B. burgdorferi* s.l.; in particular, *B. afzelii*, *B. garinii* and *B. spielmanii* DNA were detected in different tissues (blood, liver, skin) of the investigated animals [64]. Borrelia garinii, which is considered an avian-adapted spirochete, seems to cause disseminated infection in foxes more frequently than the other borrelial species known to exhibit the host specificity for mammals [65]. The same authors removed ticks from the investigated foxes and identified these arthropods as *I. ricinus*, *I. kaiseri*, *I. canisuga* and *I. hexagonus*; 32.4% of the 943 examined ticks had DNA of different borrelial species: *B. garinii*, *B. afzelii*, *B. americana*, *B. bissettiae*, *B. burgdorferi* s.s., *B. californiensis*, *B. carolinensis*, *B. lanei*, *B. spielmanii*, and *B. valaisiana* [64]. These findings suggested that the co-occurrence of different *Ixodes* species on the red foxes could increase the diversity of Borrelia species infecting the animals. In addition, *B. burgdorferi* s.l. infection was found in *I. canisuga* ticks removed from foxes in north-eastern Spain, too [66]. However, the involvement of the burrow dwelling Ixodes species in the ecology of Borrelia species and other tick-borne pathogens is limited mainly to I. hexagonus that has been demonstrated as a competent vector for Lyme disease spirochetes [67]. Infection by different genospecies of *B. burgdorferi* s.l. in foxes has been documented also in Southern Italy. In particular, six genospecies were identified in foxes’ spleens: *B. afzelii* (4/244; 1.6 %), *B. burgdorferi* s.s. (2/244; 0.8 %), *B. bissettiae* (1/244; 0.4 %), *B. garinii* (1/244; 0.4%), *B. lusitaniae* (1/244; 0.4%) and *B. valaisiana* (1/244; 0.4 %). In a single animal, a mixed infection by *B. afzelii* and *B. bissettiae* was recorded [30].

Coyotes (*C. latrans*) have been proven to be susceptible to *B. burgdorferi* infection as well. Burgess and Windberg [68] found seropositive coyotes sampled from 1984 to 1986. Furthermore, they showed that transplacental transmission can occur; in fact, they isolated *B. burgdorferi* from one coyote fetus; spirochetes were isolated also from kidneys of some adult coyotes. Coyotes also resulted in being susceptible to *B. burgdorferi* infection in Nova Scotia, Canada, based on serological and molecular analyses [69]. Even though scanty information is available about borreliosis in *C. latrans*, some studies suggested new scenarios in the role of these animals in the epidemiology of borreliosis. A new species denominated Candidatus Borrelia texasensis was isolated in 1998 from an adult male Dermacentor variabilis tick feeding on a coyote from Texas, characterized with several molecular techniques, although isolation was not possible [70]. Moreover, *Borrelia turicatae* was identified in the blood of one coyote out of 122 tested in Texas, USA [71].

Jackals seem to also be involved in the epidemiology of borreliosis. *Borrelia garinii, B. valaisiana* and *B. lusitaniae* have been detected in ticks collected from jackals in Serbia; *B. garinii* DNA was found in *D. reticulatus* whereas DNA of the other two species were detected in *I. ricinus*. Spleen samples collected from the jackals from which ticks were removed resulted in being PCR negative for *Borrelia* spp; however, the infection in the investigated animals was not excluded because spleen is not a suitable diagnostic target [42]. *Borrelia persica*, an infectious agent of the human disease TBRF (tick borne relapsing fever), has been recently found in 7.9% (5/63) of jackals (*C. aureus*) tested in Israel [72].

## 5. *Bartonella* spp.

*Bartonella* genus includes several species and subspecies, and many *Candidatus* species, most of them considered as zoonotic agents. Bartonellae are Gram-negative rods with affinity for the erythrocytes, endothelial cells and macrophages of their hosts in which sometimes can cause vasoproliferative lesions by entering endothelial cells resulting in cellular proliferation and migration [73,74].

Bartonellae are usually transmitted by hematophagous vectors. *Bartonella henselae* is well known as the etiologic agent of the human cat scratch disease and cats, usually asymptomatic hosts, are the main reservoirs whereas fleas *Ctenocephalides felis* are the main vectors. Dogs can be infected by different *Bartonella* species, such as *B. vinsonii* subsp. *berkhoffii*, *B. henselae*, *B. clarridgeiae*, *B. rochalimae*, *B. quintana*, *B. koehlerae*, *B. washoensis* and *B. elizabethae* [75]. Fleas and ticks belonging to different species are the vectors of bartonellae to dogs [76].

Some authors investigated the spreading of bartonellae also among wild canids, which resulted involved in the epidemiology of these pathogens acting as natural reservoirs. Few studies have investigated *Bartonella* infection in wolves. However, some reports showed that bartonellae can infect these canids, even though it is not clear if they can develop disease.

*Bartonella rochalimae* (previously described as *B. clarridgeiae*-like organism) has been detected in dogs, but also in wild canids such as gray foxes (U. cinereoargenteus), red foxes (*V. vulpes*) and coyotes (*C. latrans*) in California and Colorado (USA) [77,78,79]. Furthermore, DNA of *B*. *rochalimae* was found in 38.9% (42/108) of fleas (*Pulex simulans*) collected on gray foxes (*U. cinereoargenteus*) from the same geographic area [80]. Furthermore, López-Pérez et al. [81] identified *B. rochalimae* DNA in two out of 15 kit foxes (*Vulpes macrotis*) and one out of 178 coyotes from Mexico.

Pulex fleas collected on red foxes from Hungary were infected with a strain of Bartonella that was molecularly identical with *B. rochalimae* isolated from a red fox from France, suggesting that red foxes from Central Europe may also be infected with this bartonella [82]. Similarly, DNA of a *Bartonella* strain, closely related to *B. rochalimae*, was found in fleas (*Pulex irritans*) from red foxes in Andalusia, Spain [83].

Other investigations testified the circulation of *B. rochalimae* in wild canids from Europe. Its DNA was found in one *C. lupus* from Basque country, Northern Spain [84], as well as in red foxes from France and Israel [78] and in jackals from Israel [85].

*Bartonella vinsonii* subsp. *berkhoffii* has been frequently detected in dogs, but also gray and red foxes and coyotes are susceptible [77,79,81]. In addition, coyotes have been suspected of serving as wildlife reservoirs of B. vinsonii subsp. berkhoffii in some areas of California, since 28% (31/109) of tested coyotes in Santa Clara County were found to be bacteremic [86].

A serological survey carried out on island foxes (*Urocyon littoralis*) living on several islands near the Californian coast demonstrated overall seroprevalences of 25.8% (68/263) and 27.7% (73/263) for *B vinsonii* subsp. *berkhoffii* and *B. clarridgeiae*, respectively [87]. A successive investigation on island foxes from Santa Rosa Island detected 31.4% (16/51) seroprevalence for *B. clarridgeiae* only, 9.8% (5/51) for *B. vinsonii* subsp. *berkhoffii* only, and 21.6% (11/51) for both antigens [88]. Moreover, *B. vinsonii* subsp. *berkhoffii* was isolated from 11.8% (6/51) of foxes using blood culture medium and all the isolated strains resulted in belonging to the same type found in mainland gray foxes [88].

In Australia, B. clarridgeiae and *B. henseale*, usually related to cats, were detected in *C. felis* fleas removed from red foxes and *B.clarridgeiae* was also detected in blood of a red fox [89]. *B. henselae* DNA was also detected in 3 out of 20 tested arctic foxes (*Vulpes lagopus*) from Canada [90] and in spleens of two out of 142 raccoon dogs (*Nyctereutes procyonoides*) in Korea [91].

Fleishman et al. [92] investigated by indirect immunofluorescent antibody test, 97 wild canids from 19 zoos in São Paulo and Mato Grosso states, Brazil to detect antibodies against *B. henselae, B. vinsonii* subsp. *berkhoffii, B. clarridgeiae*, and *B. rochalimae* antigens. Overall, antibodies were detected in five (12.8%) of 39 crab-eating foxes (*C. thous*), three (11.1%) of 27 bush dogs (*S. venaticus*), two (8.7%) of 23 maned wolves (*Chrysocyon brachyurus*) and one (12.5%) of eight hoary foxes (*Lycalopex vetulus*), with titres ranging from 64 to 512.

Recently, wolves from Southern Italy were found harboring *Bartonella* spp. Five of six carcasses of *C. lupus* were PCR positive for these bacteria; in detail, one animal was positive for *B. rochalimae*, one for *B. vinsonii* subsp. *berkhoffii*, and one harbored DNA sequences similar to Candidatus Bartonella merieuxii clones; furthermore, one wolf was co-infected with *B.vinsonii* subsp. berkhoffii and *B. rochalimae* [93]. The detection of DNA reportable to *Candidatus* Bartonella merieuxii suggests that this new bartonella is circulating among different canids. In fact, it was previously found in dogs and jackals (*C. aureus*) from Iraq [74] and Israel [85].

The overall findings suggest that several species of wild carnivores may act as reservoirs of bartonellae. After all, *Bartonella* species are largely present among wildlife. In fact, bartonellae have been found in deer, badgers, hedgehogs and rodents in many geographic areas, as well as in different hematophagous arthropods. Therefore, wooded and semi-wooded areas are environments with features favorable to presence and spreading of different *Bartonella* species. Host adaptation of these bacteria is generally evident, in fact some species are mainly found in specific mammalian species, which represent their natural reservoirs. In the case of *B. vinsonii* subspecies berkhoffii, the host adaptation led domestic and wild canids to become the reservoirs [75]. For other species, such as *B. rochalimae*, further studies are necessary to better verify the relation with wild canids.

## 6. *Rickettsia* spp.

Genus *Rickettsia,* order Rickettsiales, family Rickettsiaceae, includes obligate intracellular small Gram-negative bacteria grouped into phylogenetic groups, namely spotted fever group (SFG), typhus group, Rickettsia bellii group, and Rickettsia canadensis group. SFG includes several species considered as pathogens of animals and humans in which cause severe diseases and usually transmitted by mites and hard ticks [94,95].

A molecular study carried out on captive red wolves (*C. rufus*) in North America did not find *Rickettsia* spp.-positive animals [96]. Conversely, different rickettsiae are circulating among wild canids in South America. A survey was carried out on maned wolf *C. brachyurus* living in the Serra da Canastra National Park (SCNP), a Cerrado preserved area in Southeast Brazil, from 2005 to 2012 [18]. Even though no blood sample from the wolves was PCR positive for *Rickettsia*, serological positive reactions were found when the serum samples from these animals were tested by indirect immunofluorescence assay versus R. parkeri, R. rickettsii, R. amblyommatis, R. rhipicephali and R. bellii antigens. In particular, 95% (74/78) of the tested wolves were reactive to at least one Rickettsia species, with R. parkeri eliciting the highest endpoint titers. Some maned wolves that were recaptured during the study were shown to seroconvert to *R. parkeri*. In addition, molecular analyses carried out on ticks removed from the same animals revealed the presence of ‘Candidatus Rickettsia andeanae’ and Rickettsia parkeri in Amblyomma tigrinum adult ticks [18].

Similar findings were reported when foxes were examined. *C. thous* foxes from Pantanal region, Brazil, were sampled in the period 2013–2015 and analyzed by serological and molecular tests; 75.6% (59/78) of these animals were seroreactive for at least one *Rickettsia* species (*R. rickettsii*, *R. parkeri*, *R. amblyommatis*) and one crab-eating fox was also PCR- positive for SFG *Rickettsia*. Among 1582 ticks, mainly *Amblyomma* spp, removed from the tested foxes, 23.5% resulted PCR positive for SFG *Rickettsia* and in 30 samples Candidatus Rickettsia andeanae was identified [97]. Moreover, Dall’Agnol et al. [98] investigated *C. thous* and *Lycalopex gymnocercus* (Pampas fox) from Brazilian Pampa and 62.5% (20/32) of the animals were seropositive for *R. parkeri*; in addition, 7.5% (22/292) of ticks collected from the tested foxes had *R. parkeri* DNA.

The pathogenicity of *R. parkeri* for domestic and wild canids remains unknown, whereas this rickettsial species is well known as the second most frequently reported human rickettsial agent in the American continent responsible for eschar-associated spotted fever [99].

The role of red foxes in the epidemiology of rickettsiosis is not fully recognized. Recently, Liu et al. [100], in China, detected *Rickettsia raoultii* in the organs (heart, liver, spleen, lungs, kidneys) of three red foxes, *Candidatus* Rickettsia barbariae in the organs of a red fox, *Rickettsia sibirica* in the liver of a red fox, and *R. raoultii* in two tick species (*Ixodes canisuga* and *D. marginatus*) from foxes.

*R. raoultii* DNA was also detected in feeding *I. ricinus* removed from wolves in Northwestern Spain [101] and in *Dermacentor reticulatus* ticks removed from a migrating golden jackal (*C. aureus*) in Denmark [102].

In Europe, since the serological surveys, red foxes from Spain resulted in being exposed to different rickettsial species. In fact, Lledó and collaborators [103] detected antibodies against *R. slovaca* and *R. typhi* in 6.7% (21/314) and 1.9% (6/314) of the analyzed foxes from Soria province, respectively. Ortuño et al. [104] found a seroprevalence of 45.9% (62/135) for *R. massiliae* and 25.2% (34/135) for *R. conorii* among red foxes from Catalona; in addition, the authors detected DNA of *R. massiliae*, *R. aeshlimannii* and *R. slovaca* in *R. sanguineus* ticks removed from the same tested foxes.

Coyotes living in the USA have been supposed to be involved in the spreading of *R. rickettsii* which is the etiologic agent of the Rocky Mountain Spotted fever in humans and dogs, endemic in many areas. Coyotes from Arizona, USA, resulted seropositive to *R. rickettsii* antigen, and PCR analyses of skin collected from the same animals showed evidence for *Rickettsia* spp. in 2.9% (4/138) of samples, even though rickettsial species were not identified [105].

Recently, *R. rickettsii* has been detected in the blood of a coyote from the US–Mexico transboundary region confirming the susceptibility of this wild canid to the pathogen [106].

Table 1 reports the cases of detection, throughout isolation or molecular methods, of bacterial arthropod-borne pathogens in relation to wild canid species.

## 7. *Leishmania* spp.

*Leishmania* spp. protozoa belong to the Trypanosomatidae family and alternate metacyclic forms (promastigotes) in Diptera (*Phlebotomus* spp. or *Lutzomyia* spp.) and intracellular stages (amastigotes) within the macrophages of mammalian hosts. Dogs are reservoir hosts of the species *Leishmania infantum* (syn. *Leishmania chagasi* in New World), and human infection occurs as infantile kala-azar and in the visceral form in endemic areas, where canids and human beings are primary hosts [107]. *Leishmania tropica* is responsible for cutaneous leishmaniasis in humans, but canine hosts seem to be involved in its life cycle in Iran, Morocco and Israel [108,109,110,111,112,113,114]. *Leishmania braziliensis* is an important etiological agent of human cutaneous leishmaniasis in Brazil [115]. The parasite has been found in dogs [116], although complex transmission cycles with sylvatic reservoir species have been suspected [117,118,119].

Systematic reviews of wild animals and canids infected by zoonotic *Leishmania* species have been recently provided [120,121]. The occurrence of such parasites in wild canids has been mostly referred to in red foxes (*V. vulpes*), probably for their taxonomic relationship with dogs [122].

Therefore, in Europe *L. infantum* has been widely reported in red foxes from France [123,124,125], Italy [17,30,126,127,128,129,130,131], Portugal [28,132,133], Spain [134,135,136,137,138,139,140,141] and Greece [142], with prevalences ranging from 0% (0/11) [140] to 59.5% (28/47) [142]. Red fox, *Vulpes corsac,* and *Vulpes zerda* were reported as incidental hosts [143]. On the other hand, the parasite was proven to occur in foxes from Brazil [144]; antibodies to *Leishmania* sp. were searched in both grey (*U. cinereoargenteus*) and red foxes in the USA [145,146]. *Leishmania infantum* was reported from foxes in Iran [147] and *Leishmania* infection in canids from Iran has been deeply revised [148,149], accounting for about 10% of positive cases in foxes.

*Leishmania* sp., presumably belonging to *L. infantum* species was found in grey foxes (*Lycalopex griseus*) from Patagonia [150]. Moreover, *Leishmania major* was reported from a fox in North Sinai [151], and in red foxes from Israel [152]. Gray foxes were serologically diagnosed as infected by *Leishmania mexicana* and *L. infantum* in Mexico [153].

The crab-eating fox (*C. thous*) in South America was considered as the main reservoir of *Leishmania donovani* [154] in Brazil; although, based on current knowledge, the parasite was probably *L. infantum*. It was reported in free-ranging animals [155] and the epidemiological role of such canid species was then reiterated [156,157] in endemic focus of *Leishmania chagasi,* where asymptomatic subjects were found infected by these protozoa. However, the role of this wildlife was debated. *Cerdocyon thous* was not able to transmit the parasites by xenodiagnosis [158] and was considered as not able to maintain the parasite life cycle, although further xenodiagnoses studies have been suggested [152,159]. Furthermore, *C. thous* was then reported as a main reservoir of the agents of American visceral leishmaniasis [160]. Some subjects from Brazil were found also infected by *Leishmania braziliensis* [161,162].

Wolves (*C. lupus*) have a closer taxonomic relationship with dogs and are considered as more suitable hosts for *Leismania* sp. compared to foxes. Most records are from Europe; the first case report of leishmaniosis in wolf was from Croatia [163], followed by several further papers dealing with epidemiological data about *L. infantum* infection in grey wolves from Spain [135,136,139,164,165,166], Portugal [167] and Italy [17,131], with prevalences ranging from 33% (34/102) [165] to 0% from 3 and 2 wolves, respectively [139,166]. However, the first report of *C. lupus* involvement in the *Leishmania* life cycle was from Iran, when *L. tropica* and *L. infantum* were isolated from the tissues of 10 wolves [111]. A mean prevalence value 10% of leishmania infection in wolves from Iran was recently reported [149].

Maned wolves (*C. brachyurus*) from Brazil have been recognized to be affected by *Leishmania* infection [155,168] and develop clinical disease, as well bush dogs (Speothos venaticus) [169], although these wild canids seem to transmit very low *L. infantum* loads to sandflies [170].

Infection in golden jackals (*C. aureus*) by *L. infantum* was first reported in Iraq [171], then pathologic features were described in an animal from Israel and serologic evidence was reported as well [24,172]. The parasite was found in jackals from Kazakhstan [173] and Georgia [174]; *L. tropica* was identified in Israel and in Algeria [152,175] while it has been reported in 10% of infected animals and 10 jackals from Iran [149]. In the last years, with the rise of the jackals’ population in Eastern Europe, infections have been reported in Serbia [176], and a subject infected by *L. infantum* was found in Romania [177].

Coyotes (*C. latrans*) were found to be infected by serology in the USA [145,146,178].

## 8. *Babesia* spp.

*Babesia* spp. are apicomplexan protozoa transmitted through tick bites, which develop by merogony into the red blood cells of a vertebrate intermediate host, where they appear as pear-shaped organisms, hence the name of piroplasms. The parasite can be transmitted by blood transfusion and, *B. gibsoni,* through wounds in fighting dogs, following saliva and blood ingestion. Dogs are infected by several *Babesia* species, referred to as large (*B. canis*, *B. vogeli* and *B. rossi*) and small (*B. gibsoni*, *B. conradae* and *B. vulpes*), with different pathogenic features [179]. The involved species in Europe are *B. vogeli*, *B. canis*, *B. gibsoni* and *B. vulpes.* Moreover, *B. gibsoni* and *B. vogeli* occur worldwide, while *B. canis* has been reported from Europe and Asia, *B. rossi* from sub-saharian Africa and *B. conradae* from the USA [180,181,182]. 

*Babesia vulpes* has recently been recognized as species nova [183], being formerly indicated as *Babesia microti-*like and *Theileria annae,* so previous records will be included referring to *B. vulpes.*

The occurrence of piroplasms in wild carnivores has been widely reported [184]. Red foxes have been found infected by *B. vulpes*, and several reports, mostly from European countries are present in the literature. *Babesia vulpes* has been reported as the sole species found in foxes from Croatia [185], Portugal [186], Italy [16,31,32,187], Hungary [188], Germany [189,190], the United Kingdom [191], Spain [144,192], Romania [193], France [125] and the Czech Republic [37]. However, *B. canis* was found in red fox from Bosnia Herzegovina [34], Austria [44], Serbia [194] and Poland [195], with a lower prevalence, always. *Babesia* spp. was then identified in 89.7% of 157 foxes in Alps [17]. *Babesia vulpes* and *B. vogeli* were found in foxes from Spain [150] and *B. vulpes* and *Babesia* sp. are reported from Slovakia [196]. *Babesia vulpes* was found along with the zoonotic species *Babesia venatorum* and *Babesia microti* in Germany [197] too. The prevalence of *B. vulpes* infection ranged from 0.98% in 205 foxes [187] to 69.2% in 63 subjects [186], with differences within the areas of the same studies, probably due to different distribution of Ixodida vectors [195]. A higher occurrence of *Babesia canis* (2.4%) was reported by Mierzejewska et al. in 383 animals [195]. *Babesia vulpes* was found in grey and red foxes from the USA and Canada [198,199], and in both Israel and Iraq [200,201], along with a new species designated as *Babesia* sp. MML related to *Babesia lengau.* Putative *Babesia* spp. were described in *Vulpes pallida* and *V. zerda* from North Africa [202]. *Babesia vulpes* and *B. vogeli* were also reported from red foxes in China [203].

Antibodies to *Babesia* sp. have not been reported in crab-eating foxes, in hoary foxes (*Lycalopex vetulus*), nor in maned wolves from Brazil, suggesting that canids of South America are not susceptible to *Babesia* spp. [204], and a study in Brazil showed serological evidence of exposure to *Babesia* spp. [205]. However, maned wolf seems to be prone to an undetermined *Babesia* sp., responsible for clinical forms [206,207]. Additionally, the DNA of a lineage strictly similar to *Babesia caballi* was identified in a crab-eating fox in Brazil [208].

Wolves too have been found infected with *Babesia,* but reports are scanty*. Babesia canis* was believed to have caused a fatal acute disease in a wolf from Hungary [209] and in Croatia [163]. Furthermore, the same parasite species was identified in several wolves, along with *Babesia capreoli* [163]. Reports from Italy refer to the occurrence of different parasite species; *B. vulpes* [187] and *B. capreoli* [17] were found in wolves from Alps, while badger-associated *Babesia* sp. badger was recently reported from Southern Italy [30].

Infected jackals with *B. canis* and *B. vulpes* were reported from Central Europe [177], *B. canis* was then identified in golden jackals from Serbia [42]. *Babesia* sp. MML was occasionally reported from Israel and Iraq [200,201], suggesting a low involvement of these canids with these hemoprotozoa. However, black-backed jackal (*C. mesomelas*) is considered the natural reservoir of *B. rossi,* the high virulent species responsible for canine babesiosis in sub-Saharan Africa [210], with 84.6% infected animals, without any overt signs of disease [211], which result in being infested by *Haemaphysalis elliptica,* the main vector of *B. rossi* [212]. Furthermore, a *Theileria* sp., very similar to *Theileria ovis,* was reported in *C. mesomelas* in a semi-arid region of South Africa [213]. *B. gibsoni* was identified in association with *Hepatozoon canis* in an Asiatic wild dog (*Cuon alpinus*) [214].

Conversely, *Lycaon pictus* (African wild dogs), found positive for *B. rossi* [215] seem to not be important reservoir hosts for the parasite [216], although a clinical case of fatal acute babesiosis in a captive animal has been described [217]. In these canids, *B. canis* was reported too [218].

Splenectomized coyotes are prone to *B. gibsoni,* after experimental inoculation [219], but intact animals develop mild clinical signs and were suggested as possible wild reservoirs of these pathogens [220]. *Babesia conradae* occurs in dogs from the USA [221]. The parasite is very similar to the morphology of *B. gibsoni* but is responsible for higher parasitemia and more pronounced anemia in dogs [222]; its transmission is still unknown. This piroplasm is also considered as emergent in coyote-hunting dogs [223,224] and although the parasite was found in the salivary glands of *Rhipicephalus sanguineus,* any attempts to transmit the infection failed [225]. On the other hand, the high prevalence in kennels with an history of coyote fighting suggested an infection route through bites and consequent bleeding [223]. Coyotes were suspected to be reservoir hosts for *B. conradae* [226], who at that time referred to the parasite as *B. gibsoni.* This hypothesis has recently been corroborated by the findings of this infection in coyotes from California [227], albeit *B. vogeli,* was also documented in coyotes [71,227].

Raccoon dogs have been found infected by *Babesia microti*-like both in South Korea [228] and in Austria [229] and by *Babesia venatorum* in Denmark [230]. Although data from Korea have not been confirmed by further investigations [231,232,233], these findings suggest an involvement of this species in the cycle of these zoonotic parasites.

## 9. *Rangelia vitalii*

*Rangelia vitalii* is a piroplasmid parasite whose life cycle is not fully elucidated and is transmitted by ticks (*Amblyomma aureolatum*). In canids, merozoites are present in red blood cells, leukocytes and endothelial cells. The protozoon occurs in South America and is responsible for anemia, bleeding, jaundice, splenomegaly and lymph nodes enlargement, mostly in young dogs. The parasite has been found in dogs in Brazil [234,235,236], Argentina [237] and Paraguay [238].

Wild canids are also infected [234], but usually asymptomatic [239], although a hemorrhagic and hemolytic syndrome has been described in crab-eating foxes [240,241]. These wild carnivores are frequently found infected, suggesting a role as carriers for the parasite [242]; parasite DNA was found in pampas foxes as well [242,243].

## 10. *Hepatozoon* spp.

*Hepatozoon* is a genus of apicomplexan parasites whose life cycle comprehends sexual stage and sporogony in an arthropod definitive host, and schizogony in various tissues of the vertebrate intermediate host. Canids are mostly infected by *Hepatozoon canis* and *Hepatozoon americanum*. The infection takes place following the ingestion of infected ticks. However, alternative modes of transmission in dogs have been identified such as vertical and trophic transmission for *H. canis* and *H. americanum,* respectively [244,245]. *Hepatozoon canis* occurs in tropical, sub-tropical and temperate climate regions of the globe [244,246], while *H. americanum* was initially found in the Southern United States [246,247]. *Hepatozoon canis* infection is often subclinical, although it may vary from asymptomatic to severe; conversely, *H. americanum* infection causes a severe disease in dogs, leading to debilitation and death.

In wild animals, *Hepatozoon* infections are usually subclinical [248,249].

In Europe, *H. canis* was found in red foxes from Spain [141,250,251], Slovakia [252], Portugal [253], Italy [17,30,32,33,254], Croatia [185], Germany [255], France [125], Hungary [51,244,256], Austria [44,189], Bosnia Herzegovina [34], the Czech Republic [37,257], Serbia [194] and Poland [195], with prevalences ranging from 5.1% to 92% in 157 and 93 *V. vulpes,* respectively [17,125]. The high frequency of infection, in areas where definitive hosts have not registered, would suggest a vertical transmission in foxes as well [44].

The parasite was found in red foxes in Israel by serology [27] and PCR [200] and in Asian wild dogs [258].

*Hepatozoon canis* was firstly recognized in a crab-eating fox in Brazil [259]. An extensive survey carried out in wild canids from North Africa allowed to identify *Hepatozoon* spp., belonging both to clade 1 (frequent in preys) and clade 2, suggesting a possible circulation of reptile parasite species, following the ingestion of paratenic hosts, preys and/or ticks infesting preys [202], to corroborate the finding of reptile-associated *Hepatozoon* lineages in crab-eating foxes from Brazil [43], then confirmed by Bazzano et al. [260]. In the same study, crab-eating foxes were found infected with a lineage very similar to *H. americanum* [43], which was reported with high prevalence along with *H. canis* in another study from Brazil [261]. *H. americanum* DNA was recovered in Uruguay from crab-eating foxes and *Lycalopex gymnocercus* (grey Patagonian fox) [262]. All these findings led to the conclusion that there is a strong association between *C. thous* and *Hepatozoon* [263].

Grey Patagonian fox has been found infected by species of *Hepatozoon* closely related to *Hepatozoon felis* [264], while in a recent study from Chile, *H. felis* was identified in Andean foxes (*Lycalopex culpaeus*), too [265]. In the same survey *H. americanum* and *H. canis* were found in American grey foxes (*Lycalopex griseus*), animal species previously positive for *H. felis-*like and *H. americanum-*like parasites [266].

The information concerning *Hepatozoon* sp. infection in wolf is very limited. To the best of our knowledge, *H. canis* was reported from Italy [17], Germany [40] and Serbia [267] with prevalences from 46% (127/276) [40] to 75% (25/33) [17].

Maned wolves from Brazil [18,268] were found infected by *H. canis*.

*Hepatozoon canis* was identified in a golden jackal (*C. aureus*) from Austria [269] and in many animals in Hungary, Israel, Iraq and North Africa [188,200,201,202].

Black-backed jackals in South Africa were recognized as affected by *H. canis* [43,213]. The parasite was recently found in a symptomatic Indian jackal (*Canis aureus indicus*) too [270].

*Lycaon pictus* were found infected by *Hepatozoon* sp. in South Africa [216,218,271], Tanzania [272] and Zambia [273].

The first case of hepatozoonosis in coyotes was documented in 1978 by Davis et al. [274], then *H. americanum* was reported in coyotes from the USA [275,276]. Coyotes were recognized to be prone to experimental infection with parasites obtained both by coyotes and dogs [277,278]. Nevertheless, a genetic diversity was observed in *Hepatozoon* spp. infecting coyotes from Oklahoma and Texas, being a major cluster related to *H. canis,* a cluster related to *H. americanum* and a third sharing features between the two groups [279].

On the other hand, an *Hepatozoon* closely related to *H. americanum*, was detected in the South American gray fox (*L. griseus*) from Argentina, and *C. thous* from Uruguay and *C. brachyurus* from Brazil [18,43,251,261,262,265,266], *Hepatozoon* spp. in bush dogs (*Speothos venaticus*) [20], and a fox of undetermined identity from Brazil [268].

Table 2 reports the cases of molecular detection of arthropod-borne protozoa in relation to wild canid species.

## 11. Conclusions

The study of the arthropod-borne pathogens spreading among wild canids is a useful tool to evaluate changes in the epidemiology of these microorganisms, which often represent a serious threat for the health of wildlife, domestic animals and humans. In fact, pathogens infecting wild canids usually can infect and cause diseases in dogs, which when share the same environments, such as in the case of hunting dogs, are largely exposed to arthropods’ bites and consequently to arthropods-borne pathogens. *Ehrlichia canis*, *A. phagocytophilum*, *B. vinsonii* subsp. *berkhoffii*, *R. rickettsii*, *R.conorii*, *Leishmania* sp., *B. canis*, *B. gibsoni*, *B. conradae*, *B. rossii*, *R. vitalii*, *H. canis* and *H. americanum* are well known as severe canine pathogens, whereas limited data about their pathogenicity for wild canids are available. Although detection of DNA and antibodies in wild hosts only shows a contact with a certain pathogen, some wild canids have been recognized to play a role as wild reservoirs of pathogens. Coyotes in North America are reservoirs for *B. gibsoni* and *B. conradae,* as well as crab-eating foxes for *R. vitalii;* raccoon dogs have been found infected by zoonotic *Babesia* species. Indeed, studies to better define the pathogenic properties of arthropod-borne pathogens for wild canids are necessary, also in view of conservation programs for canid species considered rare and even declining.

Moreover, most arthropod-borne pathogens may also infect humans, inducing diseases characterized by severe clinical forms, not always promptly recognized. Lyme disease by *B. burgdorferi*, different pathologies by several *Rickettsia* species, as well by *Leishmania* sp. and *B. venatorum* are well-known human diseases frequently reported worldwide. People living in environments with high densities of hematophagous arthropods are at high risk of infection; they can be bitten by ticks, and other arthropods, during outdoor activity for work or recreational purposes. In addition, their dogs may be attached by infected arthropods that successively can bite humans. Wild canids such as jackals, coyotes and foxes which are highly adaptable to ecosystems and human-impacted environments, are largely present in some geographic areas and are spreading across previously uncolonized areas. When they are infected by arthropod-borne pathogens, they may act as important sources of infections for humans and dogs as well as other wild animals.

In conclusion, periodic assessment of prevalences of pathogens transmitted by hematophagous arthropods in different species of wild canids should be incentivized to better determine epidemiological and pathological features of pathogens able to affect wildlife, domestic animals, mainly dogs, and humans.

## Figures and Tables

**Table 1:** Molecular or cultural detection of bacterial arthropod-borne pathogens in wild canids in relation to the animal species.

Canid Species	Common Name	Pathogens	References
*Canis aureus*	Jackal	*Anaplasma phagocytophilum*	[42]
		*Borrelia burgdorferi* s.l.	[72]
		*Bartonella rochalimae*	[85]
		*Candidatus* Bartonella merieuxii	[74,85]
*Canis latrans*	Coyote	*Ehrlichia canis*	[13]
		*Anaplasma phagocytophilum*	[41]
		*Borrelia burgdorferi* s.l.	[68,69,71]
		*Bartonella rochalimae*	[78,79,81]
		*Bartonella vinsonii* subsp. *berkhoffii*	[79,81,86]
		*Rickettsia* spp.	[105]
		*Rickettsia rickettsii*	[106]
*Canis lupus*	Grey wolf	*Ehrlichia canis*	[15,16]
		Anaplasmataceae,	[17]
		*Ehrlichia* sp.	[20]
		*Anaplasma phagocytophilum*	[39,40]
		*Borrelia burgdorferi* s.l.	[57]
		*Bartonella rochalimae*	[84,93]
		*Bartonella vinsonii* subsp. *berkhoffii*	[93]
		*Candidatus* Bartonella merieuxii	[93]
*Canis mesomelas*	Back striped jackal	*Ehrlichia canis* *Anaplasma phagocytophilum*	[22] [43]
*Cerdocyon thous*	Crab-eating fox	*Ehrlichia* sp. SFG *Rickettsia*	[19,20,21] [97]
*Chrysocyon brachyurus*	Maned wolf	*Ehrlichia canis*	[18]
*Lycaon pictus*	Lycaon	*Ehrlichia canis*	[12]
*Nyctereutes procyonoides*	Raccoon dog	*Bartonella henselae*	[91]
*Speothos venaticus*	Bush dog	*Ehrlichia* sp.	[20]
*Urocyon cinereoargenteus*	Grey fox	*Ehrlichia canis* *Anaplasma phagocytophilum* *Bartonella rochalimae* *Bartonella vinsonii* subsp. *berkhoffii*	[14] [52] [77] [77]
*Urocyon littoralis*	Island fox	*Bartonella vinsonii* subsp. *berkhoffii*	[88]
*Vulpes fulva*	American red fox	*Ehrlichia canis*	[14]
*Vulpes lagopus*	Artic fox	*Bartonella henselae*	[90]
*Vulpes macrotis*	Kit fox	*Bartonella rochalimae*	[81]
*Vulpes vulpes*	Red fox	*Ehrlichia canis*	[16,28,29,30,31,32,33]
		Anaplasmataceae	[17]
		*Candidatus* Neoehrlichia sp	[30,34,35,37]
		*Anaplasma phagocytophilum*	[31,32,33,37,44,45,46,47,48,49,50,51]
		*Borrelia burgdorferi* s.l.	[30,46,62,63,64]
		*Bartonella rochalimae*	[78,79]
		*Bartonella vinsonii* subsp. berkhoffii	[79]
		*Bartonella clarridgeiae*	[89]
		*Rickettsia raoultii*	[100]
		*Rickettssia sibirica*	[100]
		*Candidatus* Rickettsia barbariae	[100]

**Table 2:** Molecular detection of protozoal arthropod-borne pathogens in wild canids in relation to the animal species.

Canid Species	Common Name	Pathogens	References
*Canis adustus*	Side striped kackal	*Hepatozoon* sp.	[202]
*Canis aureus*	Jackal	*Leishmania infantum*	[174,176,177]
		*Leishmania tropica*	[152,175]
		*Babesia canis*	[42,177]
		*Babesia vulpes*	[177]
		*Babesia* sp. MML	[200,201]
		*Hepatozoon canis*	[188,200,201,202,269]
*Canis aureus indicus*	Indian jackal	*Hepatozoon canis*	[270]
*Canis latrans*	Coyote	*Babesia gibsoni*	[220]
		*Babesia conradae*	[221]
		*Hepatozoon* spp.	[279]
*Canis lupus*	Grey wolf	*Leishmania infantum*	[17,111,131,135,136,139,149,164,165,166,167]
		*Leishmania tropica*	[111]
		*Babesia canis*	[163,209]
		*Babesia capreoli*	[17,163]
		*Babesia vulpes*	[187]
		*Babesia* sp. badger assoc	[16]
		*Hepatozoon canis*	[17,40,267]
*Canis mesomelas*	Black-backed jackal	*Babesia rossi*	[210,211,213]
		*Theileria ovis-*like	[213]
		*Hepatozoon canis*	[43,213]
*Cerdocyon thous*	Crab-eating fox	*Leismania infantum*	[155,160]
		*Leishmania braziliensis*	[161,162]
		*Babesia caballi-*like	[208]
		*Rangelia vitalii*	[240,241,242]
		*Hepatozoon canis*	[261]
		*Hepatozoon* spp.	[202,260]
		*Hepatozoon americanum*-like	[43]
		*Hepatozoon americanum*	[262]
*Chrysocyon brachyurus*	Maned wolf	*Leishmania infantum*	[155,168]
		*Babesia* sp.	[206,207]
		*Hepatozoon canis*	[18,68]
*Cuon alpinus*	Asian wild dog	*Babesia gibsoni*	[214]
		*Hepatozoon canis*	[214]
*Lycalopex culpaeus*	Andean fox	*Hepatozoon felis*	[265]
*Lycalopex griseus*	Patagonian fox	*Leishmania* sp.	[150]
		*Hepatozoon canis*	[265]
		*Hepatozoon americanum-*like	[266]
		*Hepatozoon americanum*	[265]
		*Hepatozoon felis-*like	[266]
*Lycalopex gymnocercus*	Pampas fox	*Rangelia vitalii*	[242,243]
		*Hepatozoon americanum*	[262]
		*Hepatozoon felis-*like	[264]
*Lycaon pictus*	Lycaon	*Babesia rossi*	[215,216,217]
		*Babesia canis*	[218]
		*Hepatozoon* sp.	[216,271,272,273]
*Nyctereutes procyonoides*	Raccoon dog	*Babesia microti-*like	[229]
*Speothos venaticus*	Bush dog	*Leishmania infantum*	[169]
		*Hepatozoon* spp.	[247]
*Urocyon cinereoargenteus*	Gray fox	*Babesia vulpes*	[198,199]
*Vulpes corsac*	Corsac fox	*Hepatozoon* sp.	[202]
*Vulpes pallida*	Pale fox	*Babesia* sp.	[202]
		*Hepatozoon* sp.	[202]
*Vulpes rueppellii*	Ruppels fox	*Hepatozoon* sp.	[202]
*Vulpes vulpes*	Red fox	*Leishmania infantum*	[17,28,30,123,124,125,128,129,130,131,132,135,136,137,138,139,140,141,142,147,148,149]
		*Leishmania major*	[151,152]
		*Babesia vulpes*	[16,31,32,37,125,150,185,186,187,188,189,190,191,192,193,196,197,198,199,200,201]
		*Babesia canis*	[34,44,194,195]
		*Babesia vogeli*	[150]
		*Babesia venatorum*	[197]
		*Babesia microti*	[197]
		*Babesia* spp.	[17,196]
		*Babesia* sp. MML	[200,201]
		*Hepatozoon canis*	[17,30,32,33,34,37,44,125,141,189,195,200,254,255,256,257,258]
		*Hepatozoon* sp.	[202]
*Vulpes zerda*	Fennec fox	*Babesia* sp.	[202]
		*Hepatozoon* sp.	[202]

## Data Availability

The data presented in this study are contained within the article.
